# X-ray and computational structural study of (*E*)-2-(4-chloro­phenyl­imino­meth­yl)-4-methoxy­phenol

**DOI:** 10.1107/S1600536808023416

**Published:** 2008-07-31

**Authors:** Arzu Özek, Orhan Büyükgüngör, Çiğdem Albayrak, Mustafa Odabaşoğlu

**Affiliations:** a Department of Physics, Ondokuz Mayıs University, TR-55139 Samsun, Turkey; b Department of Chemistry, Ondokuz Mayıs University, TR-55139 Samsun, Turkey

## Abstract

In the mol­ecule of the title compound, C_14_H_12_ClNO_2_, the two aromatic rings are oriented at a dihedral angle of 5.92 (7)°. An intra­molecular O—H⋯N hydrogen bond results in the formation of a nearly planar six-membered ring, which is oriented at dihedral angles of 1.55 (4) and 5.95 (4)° with respect to the phenol and chlorophenyl rings, respectively. In the crystal structure, weak inter­molecular C—H⋯O hydrogen bonds link the mol­ecules into a three-dimensional network.

## Related literature

For related literature, see: Özek *et al.* (2007 ▶); Odabaşoğlu, Büyükgüngör *et al.* (2007 ▶); Odabaşoğlu, Arslan *et al.* (2007 ▶); Albayrak *et al.* (2005 ▶); Elerman *et al.* (1995 ▶). For general background, see: Friesner (2005 ▶); Liu *et al.* (2004 ▶).
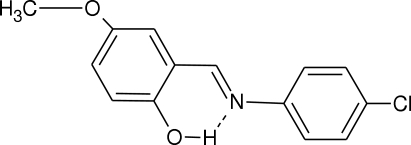


## Experimental

### 

#### Crystal data

C_14_H_12_ClNO_2_
*M* *_r_* = 261.70
Monoclinic, 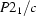

*a* = 21.2642 (19) Å
*b* = 4.7101 (3) Å
*c* = 12.2175 (12) Å
β = 93.361 (8)°
*V* = 1221.56 (18) Å^3^
*Z* = 4
Mo *K*α radiation
μ = 0.30 mm^−1^
*T* = 296 K
0.68 × 0.44 × 0.21 mm

#### Data collection

Stoe IPDSII diffractometer
Absorption correction: integration (*X-RED32*; Stoe & Cie, 2002 ▶) *T*_min_ = 0.825, *T*_max_ = 0.925
10205 measured reflections
2364 independent reflections
1789 reflections with *I* > 2σ(*I*)
*R*_int_ = 0.080

#### Refinement

*R*[*F* ^2^ > 2σ(*F* ^2^)] = 0.037
*wR*(*F* ^2^) = 0.099
*S* = 1.00
2364 reflections
167 parameters
H atoms treated by a mixture of independent and constrained refinement
Δρ_max_ = 0.20 e Å^−3^
Δρ_min_ = −0.20 e Å^−3^

### 

Data collection: *X-AREA* (Stoe & Cie, 2002 ▶); cell refinement: *X-AREA*; data reduction: *X-RED32* (Stoe & Cie, 2002 ▶); program(s) used to solve structure: *SHELXS97* (Sheldrick, 2008 ▶); program(s) used to refine structure: *SHELXL97* (Sheldrick, 2008 ▶); molecular graphics: *ORTEP-3 for Windows* (Farrugia, 1997 ▶); software used to prepare material for publication: *WinGX* (Farrugia, 1999 ▶) and *GAUSSIAN* (Frisch *et al.*, 2004 ▶).

## Supplementary Material

Crystal structure: contains datablocks I, global. DOI: 10.1107/S1600536808023416/hk2503sup1.cif

Structure factors: contains datablocks I. DOI: 10.1107/S1600536808023416/hk2503Isup2.hkl

Additional supplementary materials:  crystallographic information; 3D view; checkCIF report

## Figures and Tables

**Table 1:** Hydrogen-bond geometry (Å, °)

*D*—H⋯*A*	*D*—H	H⋯*A*	*D*⋯*A*	*D*—H⋯*A*
O1—H1⋯N1	0.88 (3)	1.79 (3)	2.6210 (18)	157 (2)
C7—H7*C*⋯O2^i^	0.96	2.56	3.495 (2)	164

Symmetry code: (i) 
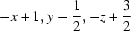
.

**Table 2:** Selected geometric parameters (Å, °) calculated with *X-RAY*, *AM1*, *PM3*, *HF* and *DFT*

Parameters	*X-RAY*	*AM1*	*PM3*	*HF*^*a*^	*DFT/B3LYP*^*a*^
C8—N1	1.276 (19)	1.292	1.302	1.262	1.293
C2—O1	1.355418)	1.368	1.357	1.336	1.344
C1—C6	1.396 (2)	1.406	1.401	1.393	1.406
C1—C8	1.448 (2)	1.466	1.459	1.467	1.449
C1—C2	1.397 (2)	1.408	1.411	1.402	1.423
N1—C9	1.418 (19)	1.409	1.431	1.408	1.406
C9—C10	1.384 (2)	1.414	1.401	1.391	1.403
C12—Cl1	1.734 (15)	1.699	1.684	1.743	1.758
C5—O2	1.3756 (18)	1.385	1.386	1.355	1.371
C11—C12—Cl1	120.72 (12)	119.860	119.505	119.595	119.538
C6—C5—O2	115.56 (14)	114.847	113.926	116.374	116.232
C6—C1—C8	119.18 (13)	116.153	118.078	118.004	119.327
C9—N1 —C8	121.22 (13)	121.780	122.176	120.342	121.253
C14—C9—N1	124.68 (13)	123.445	122.813	122.881	123.392
N1—C8—C1	122.35 (14)	123.800	119.635	123.408	122.250
N1—C9—C10	117.10 (13)	117.991	116.829	118.015	117.770
C8—C1—C2—O1	−0.9 (2)	−0.050	−0.030	−0.111	−0.085
C6—C5—O2—C7	−172.96 (15)	179.476	179.983	179.698	−179.874
C10—C9—N1—C8	−172.84 (13)	−149.450	179.999	62.793	−147.450
N1—C8—C1—C6	177.90 (14)	−177.484	−0.066	−179.307	−179.448
C1—C8—N1—C9	−178.85 (13)	−179.157	179.991	−178.540	−177.303

Notes: (a) 6-31*G*(*d*,*p*).

**Fig. 1.**
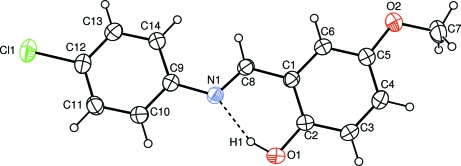
The molecular structure of the title molecule, with the atom-numbering scheme. Displacement ellipsoids are drawn at the 30% probability level. Hydrogen bond is shown as dashed line.

**Fig. 2.**
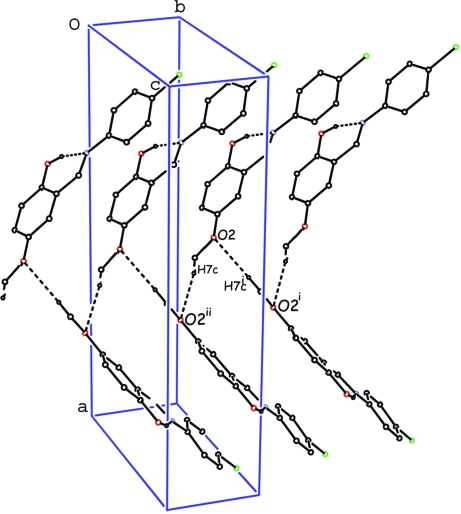
A partial packing diagram of (I) [symmetry codes: (i) 1 - x, y - 1/2, 3/2 - z; (ii) 1 - x, y + 1/2, 3/2 - z]. Hydrogen bonds are shown as dashed lines. H atoms not involved in hydrogen bonding have been omitted for clarity.

## supplementary crystallographic information

### Comment

The present work is part of a structural study of Schiff bases Özek *et al.*,  2007; Odabaşoğlu, Büyükgüngör *et al.*, 2007; Odabaşoğlu, Arslan *et al.*, 2007). We report herein the crystal structure of the title compound, (I).

In general, O-hydroxy Schiff bases exhibit two possible tautomeric forms, the phenol-imine (or benzenoid) and keto-amine (or quinoid) forms. Depending on the tautomers, two types of intramolecular hydrogen bonds are possible: O-H···N in benzenoid and N-H···O in quinoid tautomers. The H atom in (I) is located on atom O1, thus the phenol-imine tautomer is favored over the keto-amine form, as indicated by the C2-O1, C8-N1, C1-C8 and C1-C2 bonds (Fig. 1 and Table 2). The O1-C2 bond has single-bond character, whereas the N1-C8 bond has a high degree of double-bond character as in 2-(3-methoxysalicylideneamino)-1H-benzimidazole- monohydrate, (II) [where the corresponding values are C-O = 1.357 (2) Å, C-N = 1.285 (2) Å, Albayrak *et al.*,  2005]. It is known that Schiff bases may exhibit thermochromism or photochromism, depending on the planarity or non-planarity of the molecule, respectively. Therefore, one can expect thermochromic properties in (I) caused by the planarity of the molecule; the dihedral angle between rings A (C1-C6) and B  (C9-C14) is 5.92 (7)°. The intramolecular O-H···N hydrogen bond (Table 1) results in the formation of a nearly planar six-membered ring C (O1/H1/N1/C1/C2/C8), in which it is oriented with respect to rings A and B at dihedral angles of A/C = 1.55 (4)° and B/C = 5.95 (4)°. So, it is coplanar with the adjacent ring A. It generates an S(6) ring motif. The O1···N1 [2.621 (2) Å] distance is comparable to those observed for analogous ones in N-(2-hydroxyphenyl)salicylaldimine, (III) [2.675 (7) Å; Elerman *et al.*, 1995] and in three(E)-2-[(bromophenyl)iminomethyl]-4-methoxyphenols, (IV) [2.603 (2), 2.638 (7) and 2.577 (4) Å;Özek *et al.*,  2007].

In the crystal structure, weak intermolecular C-H···O hydrogen bonds (Table 1) link the molecules into a three-dimensional network (Fig. 2), in which they may be effective in the stabilization of the structure.

Ab-*initio* Hartree-Fock (HF), density-functional theory (DFT) and semi-empirical (AM1 and PM3) calculations and full-geometry optimizations were performed by means of *GAUSSIAN* 03 W package (Frisch *et al.*, 2004). The selected bond lengths and angles together with the torsion angles are compared with the obtained ones from semi-empirical, ab-initio HF and DFT/B3LYP methods (Table 2). We observe an acceptable general agreement between them. Although the DFT molecular orbital theory was considered as the most accurate method for geometry optimization for free and complex ligands (Friesner, 2005; Liu *et al.*,  2004), the HF method led to better results in regard to the bond lengths and angles.

### Experimental

The title compound was prepared by refluxing a mixture of a solution containing 5-methoxysalicylaldehyde (0.5 g 3.3 mmol) in ethanol (20 ml) and a solution containing 4-chloraniline (0.420 g 3.3 mmol) in ethanol (20 ml). The reaction mixture was stirred for 1 h under reflux. The crystals suitable for X-ray analysis were obtained from ethanol by slow evaporation (yield; 76%, m.p. 378-379 K).

### Refinement

H1 atom (for OH) was located in difference syntheses and refined isotropically [O-H = 0.88 (3) Å and U_iso_(H) = 0.112 (9) Å^2^]. The remaining H atoms were positioned geometrically, with C-H = 0.93 and 0.96 Å for aromatic and methyl H, respectively, and constrained to ride on their parent atoms with U_iso_(H) = 1.2U_eq_(C).

### Crystal data

**Table:**

C_14_H_12_ClNO_2_	*F*_000_ = 544
*M**_r_* = 261.70	*D*_x_ = 1.423 Mg m^−^^3^
Monoclinic, *P*2_1_/*c*	Mo *K*α radiation λ = 0.71073 Å
Hall symbol: -P 2ybc	Cell parameters from 10205 reflections
*a* = 21.2642 (19) Å	θ = 1.7–27.2º
*b* = 4.7101 (3) Å	µ = 0.31 mm^−^^1^
*c* = 12.2175 (12) Å	*T* = 296 K
β = 93.361 (8)º	Prismatic long stick, red
*V* = 1221.56 (18) Å^3^	0.68 × 0.44 × 0.21 mm
*Z* = 4	

### Data collection

**Table:**

Stoe IPDSII diffractometer	2364 independent reflections
Radiation source: fine-focus sealed tube	1789 reflections with *I* > 2σ(*I*)
Monochromator: plane graphite	*R*_int_ = 0.080
Detector resolution: 6.67 pixels mm^-1^	θ_max_ = 26.0º
*T* = 296 K	θ_min_ = 1.9º
ω scans	*h* = −26→26
Absorption correction: integration(X-RED32; Stoe & Cie, 2002)	*k* = −5→5
*T*_min_ = 0.825, *T*_max_ = 0.925	*l* = −14→14
10205 measured reflections	

### Refinement

**Table:**

Refinement on *F*^2^	Secondary atom site location: difference Fourier map
Least-squares matrix: full	Hydrogen site location: inferred from neighbouring sites
*R*[*F*^2^ > 2σ(*F*^2^)] = 0.037	H atoms treated by a mixture of independent and constrained refinement
*wR*(*F*^2^) = 0.099	*w* = 1/[σ^2^(*F*_o_^2^) + (0.0641*P*)^2^] where *P* = (*F*_o_^2^ + 2*F*_c_^2^)/3
*S* = 1.00	(Δ/σ)_max_ < 0.001
2364 reflections	Δρ_max_ = 0.20 e Å^−^^3^
167 parameters	Δρ_min_ = −0.20 e Å^−^^3^
Primary atom site location: structure-invariant direct methods	Extinction correction: none

### Special details

**Table:**

Experimental. 225 frames, detector distance = 120 mm
Geometry. All e.s.d.'s (except the e.s.d. in the dihedral angle between two l.s. planes) are estimated using the full covariance matrix. The cell e.s.d.'s are taken into account individually in the estimation of e.s.d.'s in distances, angles and torsion angles; correlations between e.s.d.'s in cell parameters are only used when they are defined by crystal symmetry. An approximate (isotropic) treatment of cell e.s.d.'s is used for estimating e.s.d.'s involving l.s. planes.
Refinement. Refinement of F^2^ against ALL reflections. The weighted R-factor wR and goodness of fit S are based on F^2^, conventional R-factors R are based on F, with F set to zero for negative F^2^. The threshold expression of F^2^ > 2sigma(F^2^) is used only for calculating R-factors(gt) etc. and is not relevant to the choice of reflections for refinement. R-factors based on F^2^ are statistically about twice as large as those based on F, and R- factors based on ALL data will be even larger.

### Fractional atomic coordinates and isotropic or equivalent isotropic displacement parameters (Å^2^)

**Table:**

	*x*	*y*	*z*	*U*_iso_*/*U*_eq_	
Cl1	0.03208 (2)	1.40379 (9)	0.66121 (4)	0.06631 (18)	
O1	0.26185 (6)	0.2746 (3)	0.35438 (10)	0.0677 (4)	
H1	0.2404 (12)	0.393 (5)	0.394 (2)	0.112 (9)*	
O2	0.42346 (6)	−0.2199 (3)	0.66813 (10)	0.0676 (4)	
N1	0.21631 (6)	0.5615 (3)	0.51562 (10)	0.0455 (3)	
C1	0.29911 (7)	0.2216 (3)	0.54240 (12)	0.0428 (3)	
C2	0.30182 (7)	0.1541 (3)	0.43144 (12)	0.0480 (4)	
C3	0.34597 (8)	−0.0395 (4)	0.39932 (13)	0.0575 (4)	
H3	0.3478	−0.0841	0.3254	0.069*	
C4	0.38738 (8)	−0.1674 (4)	0.47512 (14)	0.0551 (4)	
H4	0.4170	−0.2964	0.4521	0.066*	
C5	0.38493 (7)	−0.1042 (3)	0.58541 (13)	0.0493 (4)	
C6	0.34078 (7)	0.0873 (3)	0.61831 (13)	0.0484 (3)	
H6	0.3387	0.1278	0.6925	0.058*	
C7	0.47390 (9)	−0.3918 (4)	0.63688 (19)	0.0753 (6)	
H7A	0.5012	−0.2819	0.5936	0.090*	
H7B	0.4576	−0.5499	0.5945	0.090*	
H7C	0.4971	−0.4604	0.7013	0.090*	
C8	0.25442 (7)	0.4252 (3)	0.58089 (12)	0.0461 (3)	
H8	0.2534	0.4579	0.6558	0.055*	
C9	0.17376 (7)	0.7637 (3)	0.55533 (12)	0.0436 (3)	
C10	0.12943 (7)	0.8772 (3)	0.48017 (13)	0.0522 (4)	
H10	0.1291	0.8199	0.4073	0.063*	
C11	0.08576 (8)	1.0737 (3)	0.51122 (14)	0.0550 (4)	
H11	0.0561	1.1474	0.4599	0.066*	
C12	0.08649 (7)	1.1592 (3)	0.61875 (14)	0.0496 (4)	
C13	0.13070 (8)	1.0524 (4)	0.69489 (14)	0.0557 (4)	
H13	0.1312	1.1129	0.7674	0.067*	
C14	0.17395 (7)	0.8564 (3)	0.66347 (13)	0.0531 (4)	
H14	0.2037	0.7848	0.7151	0.064*	

### Atomic displacement parameters (Å^2^)

**Table:**

	*U*^11^	*U*^22^	*U*^33^	*U*^12^	*U*^13^	*U*^23^
Cl1	0.0617 (3)	0.0529 (2)	0.0864 (3)	0.01245 (18)	0.0222 (2)	0.0027 (2)
O1	0.0789 (8)	0.0846 (9)	0.0392 (6)	0.0278 (7)	−0.0008 (6)	−0.0003 (6)
O2	0.0658 (7)	0.0785 (8)	0.0577 (7)	0.0260 (6)	−0.0028 (6)	0.0012 (6)
N1	0.0478 (7)	0.0442 (6)	0.0448 (7)	0.0018 (5)	0.0049 (5)	−0.0012 (5)
C1	0.0448 (7)	0.0422 (7)	0.0418 (8)	−0.0002 (6)	0.0068 (6)	−0.0005 (6)
C2	0.0521 (8)	0.0535 (9)	0.0387 (8)	0.0048 (6)	0.0045 (7)	0.0015 (6)
C3	0.0654 (10)	0.0667 (10)	0.0414 (8)	0.0101 (8)	0.0104 (7)	−0.0051 (7)
C4	0.0548 (9)	0.0571 (9)	0.0545 (10)	0.0100 (7)	0.0114 (8)	−0.0039 (7)
C5	0.0484 (8)	0.0499 (8)	0.0495 (8)	0.0037 (6)	0.0014 (7)	0.0014 (7)
C6	0.0531 (8)	0.0507 (8)	0.0414 (8)	0.0036 (7)	0.0031 (7)	−0.0039 (7)
C7	0.0683 (11)	0.0716 (12)	0.0849 (14)	0.0251 (10)	−0.0049 (10)	0.0002 (10)
C8	0.0505 (8)	0.0467 (8)	0.0415 (8)	0.0019 (6)	0.0060 (7)	−0.0027 (6)
C9	0.0439 (7)	0.0406 (7)	0.0468 (8)	−0.0016 (6)	0.0061 (6)	−0.0005 (6)
C10	0.0572 (9)	0.0534 (9)	0.0458 (9)	0.0035 (7)	0.0021 (7)	−0.0005 (7)
C11	0.0535 (9)	0.0532 (9)	0.0579 (10)	0.0075 (7)	−0.0002 (7)	0.0058 (7)
C12	0.0464 (8)	0.0404 (7)	0.0632 (10)	−0.0002 (6)	0.0133 (7)	0.0026 (7)
C13	0.0614 (9)	0.0561 (9)	0.0500 (9)	0.0054 (7)	0.0074 (8)	−0.0058 (7)
C14	0.0548 (9)	0.0558 (9)	0.0482 (9)	0.0106 (7)	−0.0002 (7)	−0.0019 (7)

### Geometric parameters (Å, °)

**Table:**

O1—H1	0.88 (3)	C7—H7C	0.9600
C1—C6	1.396 (2)	C8—N1	1.2763 (19)
C1—C2	1.397 (2)	C8—H8	0.9300
C1—C8	1.448 (2)	C9—C10	1.384 (2)
C2—O1	1.3554 (18)	C9—C14	1.391 (2)
C2—C3	1.382 (2)	C9—N1	1.4186 (19)
C3—C4	1.379 (2)	C10—C11	1.380 (2)
C3—H3	0.9300	C10—H10	0.9300
C4—C5	1.384 (2)	C11—C12	1.373 (2)
C4—H4	0.9300	C11—H11	0.9300
C5—O2	1.3756 (18)	C12—C13	1.378 (2)
C5—C6	1.379 (2)	C12—Cl1	1.7337 (15)
C6—H6	0.9300	C13—C14	1.374 (2)
C7—O2	1.414 (2)	C13—H13	0.9300
C7—H7A	0.9600	C14—H14	0.9300
C7—H7B	0.9600		
			
C2—O1—H1	102.0 (17)	O2—C7—H7C	109.5
C5—O2—C7	117.19 (14)	H7A—C7—H7C	109.5
C8—N1—C9	121.22 (13)	H7B—C7—H7C	109.5
C6—C1—C2	118.74 (14)	N1—C8—C1	122.35 (14)
C6—C1—C8	119.18 (13)	N1—C8—H8	118.8
C2—C1—C8	122.08 (13)	C1—C8—H8	118.8
O1—C2—C3	119.19 (14)	C10—C9—C14	118.22 (14)
O1—C2—C1	121.27 (14)	C10—C9—N1	117.10 (13)
C3—C2—C1	119.54 (14)	C14—C9—N1	124.68 (13)
C4—C3—C2	121.03 (15)	C11—C10—C9	121.32 (15)
C4—C3—H3	119.5	C11—C10—H10	119.3
C2—C3—H3	119.5	C9—C10—H10	119.3
C3—C4—C5	120.05 (15)	C12—C11—C10	119.31 (14)
C3—C4—H4	120.0	C12—C11—H11	120.3
C5—C4—H4	120.0	C10—C11—H11	120.3
O2—C5—C6	115.56 (14)	C11—C12—C13	120.51 (15)
O2—C5—C4	125.11 (15)	C11—C12—Cl1	120.72 (12)
C6—C5—C4	119.33 (14)	C13—C12—Cl1	118.77 (13)
C5—C6—C1	121.29 (14)	C14—C13—C12	119.87 (15)
C5—C6—H6	119.4	C14—C13—H13	120.1
C1—C6—H6	119.4	C12—C13—H13	120.1
O2—C7—H7A	109.5	C13—C14—C9	120.76 (14)
O2—C7—H7B	109.5	C13—C14—H14	119.6
H7A—C7—H7B	109.5	C9—C14—H14	119.6
			
C6—C1—C2—O1	178.95 (14)	C14—C9—C10—C11	−1.0 (2)
C8—C1—C2—O1	−0.9 (2)	N1—C9—C10—C11	179.50 (14)
C6—C1—C2—C3	−0.9 (2)	C9—C10—C11—C12	0.3 (2)
C8—C1—C2—C3	179.23 (14)	C10—C11—C12—C13	0.6 (2)
O1—C2—C3—C4	−179.82 (16)	C10—C11—C12—Cl1	−179.55 (12)
C1—C2—C3—C4	0.1 (3)	C11—C12—C13—C14	−0.8 (2)
C2—C3—C4—C5	0.4 (3)	Cl1—C12—C13—C14	179.40 (12)
C3—C4—C5—O2	179.54 (16)	C12—C13—C14—C9	0.0 (2)
C3—C4—C5—C6	0.0 (2)	C10—C9—C14—C13	0.9 (2)
O2—C5—C6—C1	179.51 (14)	N1—C9—C14—C13	−179.70 (14)
C4—C5—C6—C1	−0.9 (2)	C1—C8—N1—C9	−178.85 (13)
C2—C1—C6—C5	1.4 (2)	C10—C9—N1—C8	−172.84 (13)
C8—C1—C6—C5	−178.79 (14)	C14—C9—N1—C8	7.7 (2)
C6—C1—C8—N1	177.90 (14)	C6—C5—O2—C7	−172.96 (15)
C2—C1—C8—N1	−2.3 (2)	C4—C5—O2—C7	7.5 (3)

### Hydrogen-bond geometry (Å, °)

**Table:**

*D*—H···*A*	*D*—H	H···*A*	*D*···*A*	*D*—H···*A*
O1—H1···N1	0.88 (3)	1.79 (3)	2.6210 (18)	157 (2)
C7—H7C···O2^i^	0.96	2.56	3.495 (2)	164

Symmetry codes: (i) −*x*+1, *y*−1/2, −*z*+3/2.

### Table 2 Selected geometric parameters (Å, °) calculated with X-RAY, AM1, PM3, HF and DFT

**Table:**

Parameters	X-RAY	AM1	PM3	HF^a^	DFT/B3LYP^a^
C8 N1	1.276 (19)	1.292	1.302	1.262	1.293
C2 O1	1.355418)	1.368	1.357	1.336	1.344
C1 C6	1.396 (2)	1.406	1.401	1.393	1.406
C1 C8	1.448 (2)	1.466	1.459	1.467	1.449
C1 C2	1.397 (2)	1.408	1.411	1.402	1.423
N1 C9	1.418 (19)	1.409	1.431	1.408	1.406
C9 C10	1.384 (2)	1.414	1.401	1.391	1.403
C12 Cl1	1.734 (15)	1.699	1.684	1.743	1.758
C5 O2	1.3756 (18)	1.385	1.386	1.355	1.371
C11 C12 Cl1	120.72 (12)	119.860	119.505	119.595	119.538
C6 C5 O2	115.56 (14)	114.847	113.926	116.374	116.232
C6 C1 C8	119.18 (13)	116.153	118.078	118.004	119.327
C9 N1 C8	121.22 (13)	121.780	122.176	120.342	121.253
C14 C9 N1	124.68 (13)	123.445	122.813	122.881	123.392
N1 C8 C1	122.35 (14)	123.800	119.635	123.408	122.250
N1 C9 C10	117.10 (13)	117.991	116.829	118.015	117.770
C8 C1 C2 O1	-0.9 (2)	-0.050	-0.030	-0.111	-0.085
C6 C5 O2 C7	-172.96 (15)	179.476	179.983	179.698	-179.874
C10 C9 N1 C8	-172.84 (13)	-149.450	179.999	62.793	-147.450
N1 C8 C1 C6	177.90 (14)	-177.484	-0.066	-179.307	-179.448
C1 C8 N1 C9	-178.85 (13)	-179.157	179.991	-178.540	-177.303

Notes: (a) 6-31G(d,p).
